# Thermal-State Continuous-Variable Quantum Key Distribution Under the Effects of Gravity

**DOI:** 10.3390/e28050501

**Published:** 2026-04-28

**Authors:** Li Zhang, Jiannan Huang, Jian Zhou

**Affiliations:** 1 School of Software, Changsha Social Work College, Changsha 410004, China; 2 College of Computer and Mathematics, Central South University of Forestry and Technology, Changsha 410004, China

**Keywords:** continuous variable quantum key distribution, thermal-state, gravity

## Abstract

Continuous-variable quantum key distribution has gradually shifted from optical fiber communication to space communication. In free-space quantum communication, the influence of gravity cannot be ignored. In light of the influence of gravity, this study assesses the efficacy of the thermal-state continuous-variable quantum key distribution (QKD) protocol in a non-inertial reference frame. This differs from the conventional scenario in an inertial reference frame, where pure vacuum Gaussian states are employed without consideration of the influence of gravity. This study examines the potential and challenges of quantum key distribution in the presence of gravitational effects. The feasibility of generating the key under the influence of gravity through the lens of quantum state transfer in a non-inertial reference system is also analyzed. It presents a comprehensive mathematical derivation and simulation of the secret key rate for maintaining a positive rate under specific conditions. Furthermore, it presents a detailed implementation plan for thermal-state quantum key distribution in a non-inertial reference frame, particularly in the context of gravity. It offers valuable insights into the performance of quantum communication in unconventional settings.

## 1. Introduction

Quantum key distribution (QKD) [1,2] provides two distant parties, Alice and Bob, with a means of securely transmitting information over an insecure quantum channel. The foundations of QKD are rooted in fundamental physical principles [3], including the non-cloning theorem and the Heisenberg uncertainty principle. QKD establishes a shared key between the two distant communication parties, which can only be obtained by the legitimate participants. It employs quantum bits as the information carriers to achieve efficient encryption and transmission through the utilization of quantum superposition and quantum entanglement [4]. The original QKD employed optical fiber as the transmission medium. With the continuous advancement of technology, fiber-optic QKD is gradually evolving towards free-space communication [5,6].

Most QKD protocols can be classified as either a discrete-variable (DV) protocol [7,8] or continuous-variable (CV) protocol [9,10,11,12], based on how classical information is being encoded. Compared with DV-QKD, CV-QKD can take advantage of the continuous parameters of the light field, such as phase and amplitude, to encode information, and more keys can be transmitted at the same time, which is more advantageous in high-speed data transmission and metropolitan area scenarios [13,14]. The implementation cost is also further reduced due to the relative maturity of the optics required for the CV-QKD scheme [15]. Using a continuous-variable system can yield significant advantages [16,17]. In Gaussian-modulation quantum key distribution, Alice prepares a lot of pure vacuum states for source. However, this is technically impossible to achieve as small amounts of unknown Gaussian preparation noise can not be avoided. Under the circumstances, it is of great importance to analyze the security of thermal-state quantum key distribution [18,19,20].

QKD has undergone considerable advancement, progressing from theoretical concepts to tangible applications. The launch of the “Micius” satellite [21] in 2016 marked the instance of satellite-based quantum communication. Subsequently, CV-QKD technology continued to enhance efficiency and security through optimized information coordination [22], the introduction of multidimensional reconciliation virtual channels [23], an analysis of the impact of code word length [17], and the adoption of adaptive coordination protocols. These developments illustrate the accelerated evolution of QKD and its potential to enhance network security.

The field of quantum technology has recently expanded to include satellite-based communications [24,25], which has led to a need for a reassessment of quantum information protocols to account for the impact of gravitational fields on quantum states [26,27]. This paper addresses the pivotal question of how gravity, particularly through acceleration effects, affects CV-QKD protocols [26,28,29]. Moreover, this knowledge is extended to scenarios where both Alice and Bob are affected by acceleration, which is crucial for the practical applications of quantum networks. For the sake of analyzing the effect of gravity, a generalized Rindler frame of reference is studied and the corresponding state transformation is considered as a Gaussian channel [30]. In contrast with the conventional assumption of ideal initial states in earlier CV-QKD protocols, this paper considers a more realistic scenario using the thermal state, thereby acknowledging the experimental imperfections inherent in the practice. A detailed derivation of formulas and an analysis of simulation diagrams demonstrate that thermal-state CV-QKD can achieve positive key rates even when subjected to the effects of gravity [30,31]. This study advances the theoretical framework of CV-QKD and provides crucial insights for the practical implementation and optimization of quantum communication systems in dynamic gravity environments.

The organizational structure of this article is as follows. Section 2 introduces the thermal-state CV-QKD affected by gravity while Section 3 derives the secret key rate of the thermal-state CV-QKD under the influence of gravity. In Section 4, a simulation and performance analysis is conducted to evaluate the protocol’s performance and explain the protocol based on actual parameters, including shot noise, variance, and excess noise. The summary is given in Section 5.

## 2. Thermal-State Continuous-Variable Quantum Key Distribution Under the Effects of Gravity

This section addresses the impact of gravity on the thermal-state CV-QKD protocol. Following the equivalence of Schwarzschild and Rindler metrics in the near-horizon limit, the impact of gravity on the quantum state can be regarded as analogous to the influence of the observer’s acceleration on the quantum state. This can be quantified and elucidated by comparing the covariance matrix in inertial and non-inertial reference frames. The gravitational field alters the characteristics of the quantum state and the covariance matrix of the two local wave packets. This alteration will impact the efficacy of QKD protocols, particularly regarding the secret key rate. The equivalence principle and alterations in the covariance matrix can be employed to ascertain and elucidate the influence of gravity on the quantum key distribution protocol. The impact of gravity can be conceptualized as a curved dynamic background, a perspective that modifies the formulae of quantum field theory and has significant ramifications for foundational quantum mechanics.

In the general CV-QKD protocol, it is assumed that Alice sends a large number of pure vacuum states. However, this is only an idealization, which is not true in practice, because there is always a small amount of unknown Gaussian preparation noise. It should be noted that the noise is trusted noise which can not be used by Eve to recover information [32]. Considering that, it is significant to study the scenarios in which the protocol uses Gaussian thermal states. Using thermal states enhances system security by adding more noise, making it difficult for attackers to obtain information without detection. Additionally, thermal states are easier to implement in experiments, can reduce technical complexity and cost, and are compatible with existing communications infrastructure. The study of thermal-state CV-QKD contributes to a deeper comprehension of the behavior of quantum systems in non-ideal circumstances; enhances the resilience of the system to noise-induced errors; and, in certain instances, improves the key rate, optimizes the utilization of quantum resources, and propels the advancement of quantum communication technology towards a more practical and commercial orientation. The quantum states originally prepared by Alice can be described by the Heisenberg picture as $\hat{X_{A}}={\hat{X}}_{s}+\hat{X_{0}}$, where ${\hat{X}}_{s}$ refers to the classical signal and $X_{0}$ is the quantum noise of the thermal mode. The total variance of Alice’s states is

(1) $$\hat{V}={\hat{V}}_{s}+{\hat{V}}_{0}.$$

The variance of the thermal states can be further decomposed into the variance of the pure vacuum state (usually normalized to 1) and the random noise ζ of Alice’s square, ${\hat{V}}_{0}=1+\zeta$. In general, we simply have $\hat{V}={\hat{V}}_{s}+1$, and the preparation noise ζ is treated as 0. This paper analyzes the influence of non-zero preparation noise on Alice model preparation. It is assumed that Eve does not control the preparation noise.

Eavesdropping attacks can be classified into two main categories [33]: individual attacks (IAs) and collective attacks (CAs). In the case of an individual attack, the stolen states are measured independently. Consequently, the probability distribution of the classical symbols that Alice and Bob had previously shared was preserved. In a collective attack, the adversary, Eve, maintains her auxiliary devices in quantum storage until the classical post-processing process is complete and optimal collective measurements have been performed. The impact of attack modes on CV-QKD schemes varies, particularly in terms of key rates. The performance of the CV-QKD protocol is analyzed in accordance with the various attack modes. It is often assumed that the eavesdropper, Eve, performs a collective Gaussian attack, which is one of the most powerful attacks in quantum physics. The entanglement clone represents the most significant and practically relevant example of this phenomenon and will be employed in our analysis. Upon examination of Eve’s behavioral patterns, it becomes evident that she replaces the authentic connection between Alice and Bob with a singular operation that effectively simulates the channel between them. This operation can be represented by a thermal noise channel with a transmittance T, which can be practically achieved by a beam splitter with a parameter T.

**Figure 1:**
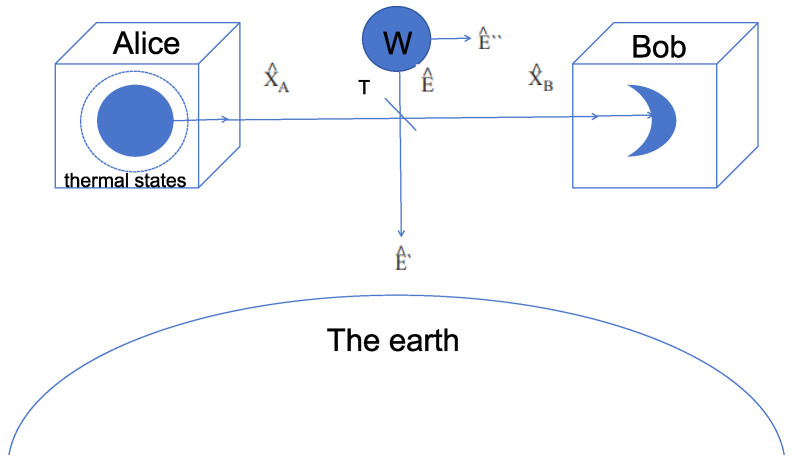
Scheme of a CV-QKD protocol using thermal states under the effect of gravity. A beam splitter with channel transmission T models the loss in the quantum channel. The eavesdropping attack is a Gaussian collective attack in the form of an entangling cloner attack where the variance of the Einstein–Podolsky–Rosen (EPR) state is W with the modes of the EPR beam described by the operators ${\hat{E}}^{\prime \prime }$ and ${\hat{E}}^{\prime }$. The initial mode sent by Alice ${\hat{X}}_{A}$ is in a thermal state and once Bob receives the mode ${\hat{X}}_{B}$ he will perform either a homodyne or heterodyne measurement on it.

The specific steps of the thermal-state CV-QKD protocol in the standard non-inertial frame are as follows (see Figure 1):

Step 1: Alice prepares an EPR state with variance V and sends one of the modes to Bob through a quantum channel in the non-inertial frame, who randomly measures their share of entangled states; the channel has a transmission T and excess noise ϵ. In this context, the variable χ represents the noise concerning the channel’s input.

(2) $$\chi =\frac{1}{T}+\epsilon -1.$$

Step 2: They carried out a procedure to shift the data, discarding the measurements taken on different substrates. Eve, an eavesdropper, interacts with the quantum system to learn the key. However, her actions change the state in a detectable way due to the No-cloning theorem.

Step 3: They perform a parameter estimation, and the reliable parts reveal randomly selected data samples to each other to estimate the parameters of the channel and thus the data obtained by Eve. After the above two steps, Alice and Bob share a data string called the raw key.

Step 4: They use classical communication methods to get a universal binary key from the data. This protocol is called direct or reverse reconciliation depending on whether Alice’s or Bob’s data is used. Specifically, when Bob or Alice attempts to estimate the result measured by Alice or Bob, this protocol is called direct or reverse reconciliation. Therefore, Alice or Bob is the direct or reverse reconciliation reference point.

Step 5: They perform error correction and privacy amplification to obtain relevant keys.

## 3. Security Analysis

To determine whether the set of binary symbols created in the protocol can be used to encrypt information, two honest parts must estimate the secret key rate. The calculations are limited to reverse reconciliation, as direct reconciliation can not beat the 3 dB loss limit. The secret key rate for reverse reconciliation is defined as follows:

(3) K=βI(a:b)−S(b:E).

Here, β is the negotiation efficiency. I(a:b) is the Shannon entropy between Alice and Bob while S(b:E) denotes the von Neumann entropy between Bob and Eve. In the EB scenario, Alice prepares an EPR state of zero mean and covariance matrix as Equation (4).

(4) $$\sigma_{AA^{\prime }}=\left[\begin{array}{cc} VI & \sqrt{V^{2}-1}\sigma_{z}\\ \sqrt{V^{2}-1}\sigma_{z}\; & VI \end{array}\right],$$

The parameters in the above Equation (4) are as

(5) $$\sigma_{z}=\left(\begin{array}{cc} 1 & 0\\ 0 & -1 \end{array}\right),$$

(6) $$I=\left(\begin{array}{cc} 1 & 0\\ 0 & 1 \end{array}\right).$$

After one mode is sent to Bob through the noisy channel introduced by Eve, the covariance matrix of the output can be easily computed.

(7) $$\sigma_{AB}=\left[\begin{array}{cc} xI & z\sigma_{z}\\ z\sigma_{z} & yI \end{array}\right],$$

and the parameters used in Equation (7) are as follows:

(8) $$\begin{array}{cc} x & =V,\\ y & =T(V+\chi ),\\ z & =\sqrt{T(V^{2}-1)}. \end{array}$$

Considering the thermal-state resource, the following covariance matrix is given.

(9) $$\sigma_{AB}^{\left(t\right)}=\left[\begin{array}{cc} uI & w\sigma_{z}\\ w\sigma_{z} & vI \end{array}\right],$$

and the parameters used above are as follows:

(10) $$\begin{array}{cc} u & =V+1,\\ v & =TV+(1-T)W,\\ w & =\sqrt{T{\left(V_{s}+1\right)}^{2}-1}. \end{array}$$

The above calculation results are obtained under the initial frame and the extension to the non-initial frame is given in the following.

The following examines the effect of uniform acceleration on the Gaussian-modulation quantum key distribution protocol. This is done by studying the effect of uniform acceleration on a two-mode Gaussian state. An equivalence has been built that transforming any two-mode Gaussian state of two localized wave packets to an accelerated frame of reference corresponds to a pair of uniformly accelerated observers. In doing so, the transformation can be represented as a Gaussian channel. The equivalence principle is used to consider two observers accelerating uniformly. A two-mode state is first prepared in an inertial system. The state in the inertial frame is transformed to a non-initial frame by Bogoliubov transformations.

A quantum field adhering to the Klein–Gordon equation (see Equation (11)) is under discussion. This equation serves as a foundational description for a real, massive, scalar quantum field.

(11) $$\begin{array}{c} \left(□+m^{2}\right)\hat{\Phi }=0. \end{array}$$

The field operator $\hat{\Phi }$ can be expressed in terms of orthogonal solutions to the equation. The field operators obey bosonic commutation relations. The discussion focuses on two distinct decomposition methods, one suited for Minkowski observers in an inertial reference frame and the other for Rindler observers in a uniformly accelerated reference frame. These two operations transform Gaussian states into Gaussian states. Under the circumstance, the effect of relativistic acceleration on localized two-mode Gaussian quantum states is studied.

In the initial method, the mode functions utilized are $\phi_{k}$, which encompass solely the positive frequency components associated with the time-like Killing vectors in Minkowski spacetime. In the second method, the mode functions employed are $\psi_{k}$, which include only the positive frequency components related to the time-like Killing vectors in Rindler spacetime. The annihilation operators are denoted by ${\hat{f}}_{k}$ and ${\hat{d}}_{k}$, respectively. Consequently, the expression for the field operator can be reformulated for these two different scenarios.

(12) $$\begin{array}{c} \hat{\Phi }=\sum_{k}\phi_{k}{\hat{f}}_{k}+H.C.=\sum_{k}\psi_{k}{\hat{d}}_{k}+H.C. \end{array}$$

The field operator $\hat{\Phi }$ can be decomposed into the Minkowski modes $u_{k}$ and the corresponding annihilation operators ${\hat{a}}_{k}$ obey canonical communication relations $\left[{\hat{a}}_{k},{\hat{a}}_{l}\right]=\left[{\hat{a}}_{k}^{†},{\hat{a}}_{l}^{†}\right]=0$ and $\left[{\hat{a}}_{k},{\hat{a}}_{l}^{†}\right]=\delta \left(k-l\right)$.

It is now assumed that, in the initial state, only two modes, $\phi_{I}$ and $\phi_{II}$, are occupied, with corresponding annihilation operators ${\hat{f}}_{I}$ and ${\hat{f}}_{II}$. Similarly, we consider only two $\psi_{k}$: $\psi_{I}$ and $\psi_{I}$, and annihilation operators ${\hat{d}}_{I}$ and ${\hat{d}}_{II}$. The remaining $\psi_{k}$ is not empty, but we assume that each accelerated observer can access only one mode. State transitions from one frame of reference to another are noisy Gaussian channels. To write the action of this channel, the quadrature operators ${\hat{q}}_{\Lambda }^{\left(f\right)}$ and ${\hat{p}}_{\Lambda }^{\left(f\right)}$ associated with $\phi_{\Lambda }$ is introduced, where Λ∈{I,II}. With the help of Equation (12), the Bogoliubov transformation relating the two sets of creation and annihilation operators can be obtained. We follow the definition in [34],

(13) $${\hat{d}}_{\Lambda }=\sum_{k}\left[\left(\psi_{\Lambda },\phi_{k}\right){\hat{f}}_{k}+\left(\psi_{\Lambda },\phi_{k}^{*}\right){\hat{f}}_{k}^{†}\right].$$

This equation is very important to help us to calculate the transformational matrix *M*. On the other hand, the bosonic operator ${\hat{f}}_{k}$ can be given after taking the scalar product $\left(\phi_{\Lambda },•\right)$ into account.

(14) $${\hat{f}}_{\Lambda }=\int dk\left(\phi_{\Lambda },u_{k}\right){\hat{a}}_{k}+\left(\phi_{\Lambda },u_{k}^{*}\right){\hat{a}}_{k}^{†}.$$

Similarly, the quadrature operators associated with $\psi_{\Lambda }$ are defined as ${\hat{q}}_{\Lambda }^{\left(d\right)}$ and ${\hat{p}}_{\Lambda }^{\left(d\right)}$.

(15a) $${\hat{q}}_{\Lambda }^{\left(f\right)}=\frac{{\hat{f}}_{\Lambda }+{\hat{f}}_{\Lambda }^{†}}{\sqrt{2}},$$

(15b) $${\hat{p}}_{\Lambda }^{\left(f\right)}=i\frac{{\hat{f}}_{\Lambda }^{†}-{\hat{f}}_{\Lambda }}{\sqrt{2}}.$$

The related quadratures should be combined into a single vector as follows:

(16) $$\begin{array}{c} {\vec{x}}^{\left(i\right)}=\left\{{\hat{q}}_{I}^{\left(i\right)},{\hat{p}}_{I}^{\left(i\right)},{\hat{q}}_{II}^{\left(i\right)},{\hat{p}}_{II}^{\left(i\right)}\right\}. \end{array}$$

where i∈{I,II}. Finally, the Bogoliubov transform can be expressed as follows:

(17) $$\begin{array}{c} X^{\left(d\right)}=MX^{\left(f\right)}, \end{array}$$

(18) $$\begin{array}{c} \sigma^{\left(d\right)}=M\sigma^{\left(f\right)}M^{T}+N. \end{array}$$

where M and N are 4 × 4 real matrices and N describes the noise present in the quantum channel [34]. We define the following:

(19a) $$\begin{array}{cc} \alpha_{\Lambda } & =(\Psi_{\Lambda },\phi_{\Lambda }), \end{array}$$

(19b) $$\begin{array}{cc} \beta_{\Lambda } & =-(\Psi_{\Lambda },\phi_{\Lambda }^{*}). \end{array}$$

In scenarios where the squeezing parameters associated with the channel inputs are of considerable magnitude, the $\beta_{\Lambda }$ coefficients may be considered negligible, as they are substantially less significant, being several orders of magnitude smaller than the $\alpha_{\Lambda }$ coefficients. Under this approximation, the matrix can be reduced to the following form:

(20) $$\begin{array}{c} M=\alpha_{I}I⨁\alpha_{II}I, \end{array}$$

The N matrix is given by

(21) $$\begin{array}{c} N=\left(1-\alpha_{I}^{2}\right)I⨁\left(1-\alpha_{II}^{2}\right)I. \end{array}$$

The I matrix is as follows:

(22) $$I=\left(\begin{array}{cc} 1 & 0\\ 0 & 1 \end{array}\right).$$

The mode functions, $\phi_{\Lambda }$ and $\psi_{\Lambda }$, are chosen such that their respective states can be prepared and quantitatively assessed utilizing apparatuses with finite dimensions. This necessitates that the wave packets are effectively localized and consist predominantly of positive frequency components centered around the spectral centroid $\Omega_{0}$. Additionally, $\Omega_{0}$ must satisfy $\Omega_{0}\gg 1/L$ to minimize the contribution of negative frequencies as much as is feasible. Moreover, the mode function $\psi_{\Lambda }$, which is subject to acceleration, must be positioned at a substantial distance from the event horizon when considered concerning its characteristic length scale, L. Then, we denote the proper acceleration at the center of $\psi_{\Lambda }$ by $A_{\Lambda }$. The proper acceleration on $\psi_{\Lambda }$ is approximately constant if the requirement, $1/A_{\Lambda }\ll L$, is satisfied. It should be stressed that individual points of any finite-size detector that undergoes a uniformly accelerated motion must experience different proper accelerations. The hyperbolae in the Rindler chart are characterized by proper accelerations ranging from zero to infinity. To assign an approximate single proper acceleration A to an extended body, one should ensure that its proper length *L* is sufficiently small as compared to its proper distance from the event horizon, 1/A<<L.

This allows for the attribution of $A_{\Lambda }$ to $\psi_{\Lambda }$ as the value of the proper acceleration on the latter. We first define the mode functions and their first derivatives on the Cauchy surface t=η=0. The mode function $\phi_{\Lambda }$ satisfies the initial conditions,

(23a) $$\begin{array}{c} \phi_{\Lambda }\left(x,0\right)=\pm Ce^{-2{\left(\frac{x_{0}}{L}\log\left(\frac{\chi }{x_{0}}\right)\right)}^{2}}sin\left(\sqrt{\Omega_{0}^{2}-m^{2}}\left(x-x_{0}\right)\right), \end{array}$$

(23b) $$\begin{array}{c} \partial_{t}\phi_{\Lambda }\left(x,0\right)=-i\Omega_{0}\phi_{\Lambda }\left(x,0\right), \end{array}$$

where $x_{0}$ represents the position of the center point of the function, L denotes the width of the wave packet, C is the normalization constant, and the lower sign correlates with the value of λ, which is equal to I(II). The envelope is maintained constant, with trigonometric functions replaced by modified Bessel functions and inertial coordinates substituted for Rindler coordinates. In a 1 + 1-dimensional spacetime, the Rindler coordinates describe an accelerated reference frame (η, χ).

(24a) $$\begin{array}{c} t=\chi sinh(a\eta ), \end{array}$$

(24b) $$\begin{array}{c} x=\chi cosh(a\eta ). \end{array}$$

In this context, *a* denotes acceleration and (t, x) represents the coordinates within the Minkowski coordinates. In Rindler coordinates, the mode functions are given by

(25a) $$\begin{array}{c} \psi_{\Lambda }\left(\chi ,0\right)=C^{\prime }e^{-2{\left(\frac{x_{0}}{L}\log\left(\frac{\chi }{x_{0}}\right)\right)}^{2}}\times G\left(\chi \right). \end{array}$$

(25b) $$\begin{array}{c} \partial_{t}\psi_{\Lambda }\left(\chi ,0\right)=\mp i\Omega_{0}\psi_{\Lambda }\left(\chi ,0\right). \end{array}$$

where G(χ) can be expressed as follows,

(26) $$\begin{array}{c} G\left(\chi \right)=ℑ\{I_{\frac{-i\Omega_{0}}{A}}\left(m\right|x_{0}\left|\right)I_{\frac{i\Omega_{0}}{A}}(m|\chi |)\}. \end{array}$$

In this context, $C^{\prime }$ represents a normalization constant, while $I_{iv}\left(x\right)$ denotes the modified Bessel function of the first kind. The CVQKD protocol is formulated in terms of localized field modes.

The operation of transforming a Gaussian state from a non-initial frame to an initial one can be regarded as the transmission of a noisy Gaussian channel. Combining Equations (20) and (21), the transformation of the statistical moments of a general Gaussian state under the action of the channel with relativistic acceleration reads as

(27) $$\begin{array}{cc} \; & \sigma_{AB}^{\left(d\right)}=M\sigma_{AB}^{\left(t\right)}M^{T}+N,\\ \; & X^{\left(d\right)}=MX^{\left(t\right)}. \end{array}$$

Here, *M* and *N* are 4×4 real matrices and the matrix *N* corresponds to the noise present in the quantum channel while *M* is the transformation matrix decided by acceleration.

Affected by the gravity, the covariance matrix changes into Equation (28):

(28) $$\sigma_{AB}^{\left(d\right)}=\left[\begin{array}{cc} rI & t\sigma_{z}\\ t\sigma_{z}\; & sI \end{array}\right],$$

where r, s, and t are given by

(29) $$\begin{array}{cc} r & =\alpha_{I}^{2}x+\left(1-\alpha_{I}^{2}\right),\\ s & =\alpha_{II}^{2}y+\left(1-\alpha_{II}^{2}\right),\\ t & =\alpha_{I}\alpha_{II}z. \end{array}$$

The quantities $\alpha_{I}$ and $\alpha_{II}$ are the Bogoliubov coefficients computed in [35]. In this paper, the reverse reconciliation is considered as the direct reconciliation can not break the 3 dB limit. Then, the secret key rate under gravity is analyzed. Affected by the gravity, the I(a:b) changes for coherent states with homodyne detection which is given by

(30) $$I\left(a:b\right)=\frac{1}{2}\log_{2}\left(\frac{r+1}{r+1-t^{2}/s}\right).$$

And for heterodyne detection it is

(31) $$I\left(a:b\right)=\frac{1}{2}\log_{2}\left(\frac{r+1}{ur+1-t^{2}/\left(s+1\right)}\right).$$

These previous equations are derived by the $I\left(a:b\right)=\frac{1}{2}\log_{2}\left(\frac{a+1}{a+1-c^{2}/b}\right)$.

For collective attacks, the Holevo bound in reverse reconciliation is given by

(32) S(b:E)=S(E)−S(E∣b).

Assuming that Eve holds the purification of the state allows us to write Equations (33) and (34), and Eve’s accessible information is a function of the entropic quantities of Alice and Bob only.

(33) S(E)=S(AB),

(34) S(E∣b)=S(A∣b).

The parameters above also can be expressed as

(35) $$S\left(AB\right)=g\left(\lambda_{1}\right)+g\left(\lambda_{2}\right),$$

(36) $$S\left(A|b\right)=g\left(\lambda_{3}\right)+g\left(\lambda_{4}\right),$$

where $\lambda_{1,2}$ are the symplectic eigenvalues of Equation (28), given by

(37) $$\lambda_{1,2}^{2}=\frac{1}{2}\left(▵\pm \sqrt{{▵}^{2}-4D^{2}}\right).$$

In the above equation, the parameters ▵ and *D* [13] are

(38) $$▵=r^{2}+s^{2}-2t^{2},$$

(39) $$D=rs-t^{2},$$

And g(x) can be expressed as follows:

(40) $$g\left(x\right)=\left(\frac{x+1}{2}\right)\log_{2}\left(\frac{x+1}{2}\right)-\left(\frac{x-1}{2}\right)\log_{2}\left(\frac{x-1}{2}\right).$$

Additionally, $\lambda_{3,4}$ are as follows:

(41) $$\lambda_{3,4}^{2}=\frac{1}{2}\left(A\pm \sqrt{A^{2}-4B}\right),$$

The parameters in Equation (41) are as follows:

(42) $$A=\frac{1}{r+1}\left[r+sD+▵\right],$$

(43) $$B=\frac{D}{r+1}\left[s+D\right].$$

## 4. Simulation and Performance Analysis

Before discussing in detail the effect of gravity on the protocol under different values of parameters, the relationship between channel losses l and transmission T is defined as follows:

(44) $$T=10^{-l/10}.$$

The total variance of the modes initially prepared by Alice is expressed as $V=V_{S}+V_{0}$, where $V_{S}$ represents the variance of modulation which obeys Gaussian distribution with a mean of zero while $V_{0}$ denotes the variance of shot noise. Within the analysis, the scenario is examined in which the variable $V_{0}$ is not inherently equivalent to one. To comprehensively investigate the influences on the performance of the thermal-state CV-QKD protocol in the presence of gravity, the analysis is extended to include the shot noise $V_{0}$, the variance of the EPR-entangled Gaussian state W prepared by Alice, and the Bogoliubov coefficients $\alpha_{I}$ and $\alpha_{II}$, as well as the excess noise ϵ. The results are visualized in the following figures.

**Figure 2:**
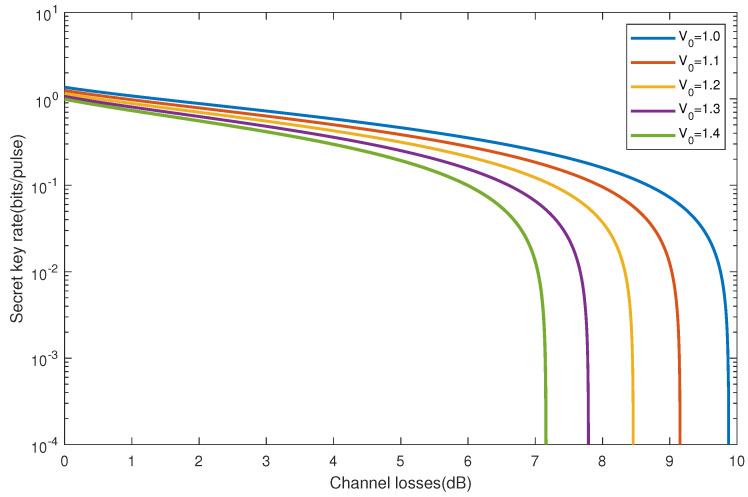
Secret key rate for different shot noise $V_{0}$ as a function of channel losses are shown. From top to bottom (right to left) are 1, 1.1, 1.2, 1.3, and 1.4, respectively. The parameters: W = 1, $\alpha_{I}$ = 0.985, $\alpha_{II}$ = 0.985, and ϵ = 0.

Figure 2 illustrates the relationship between channel loss and secret key rate at different levels of shot noise $V_{0}$. The curve decreases with the increase of $V_{0}$, which indicates that channel loss hurts the key rate. High $V_{0}$ values can significantly reduce the key rate even at low channel losses. The curve drops rapidly from left to right when $V_{0}$ = 1.4. It drops more slowly when $V_{0}$ = 1. Different $V_{0}$ values have different sensitivity to channel loss. As the channel loss increases (on the X-axis), the slopes of all curves become steeper, indicating that channel loss has a linear effect on the key rate. Even if the $V_{0}$ value is low, the key rate will drop dramatically if the channel loss is large enough. With the increase of $V_{0}$, the influence of channel loss on the key rate becomes more obvious, resulting in a general decrease in the key rate. Under different $V_{0}$ values, the channel loss has various effects on the key rate, but it is generally a negative correlation.

**Figure 3:**
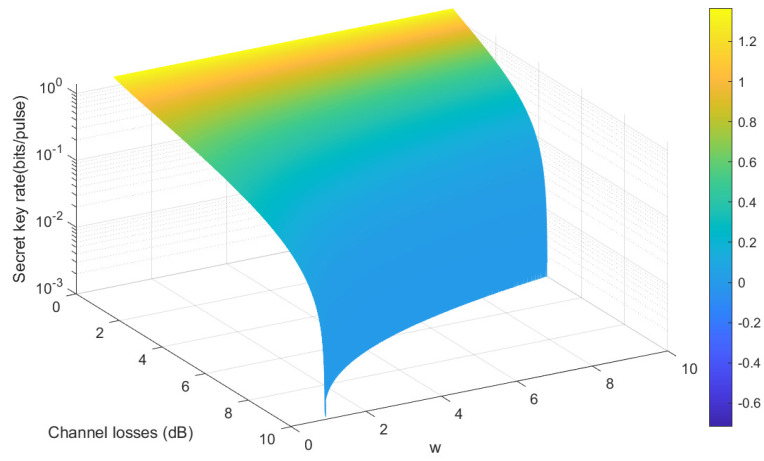
Secret key rate for different variance W of Eve’s ancilla modes in an EPR-entangled Gaussian state as a function of channel losses is shown. The parameters: $V_{0}$ = 1.1, $\alpha_{I}$ = 0.985, $\alpha_{II}$ = 0.985, ϵ = 0, and β=0.98.

Figure 3 illustrates the relationship between channel losses, variance W, and secret key rate. Color transitions from yellow to blue, representing different secret key rates. As channel losses increase, the curve shows a downward trend, and when the value of W is high, the curve is steeper, indicating that even at high channel losses, a higher key rate can be achieved with a low W value.

**Figure 4:**
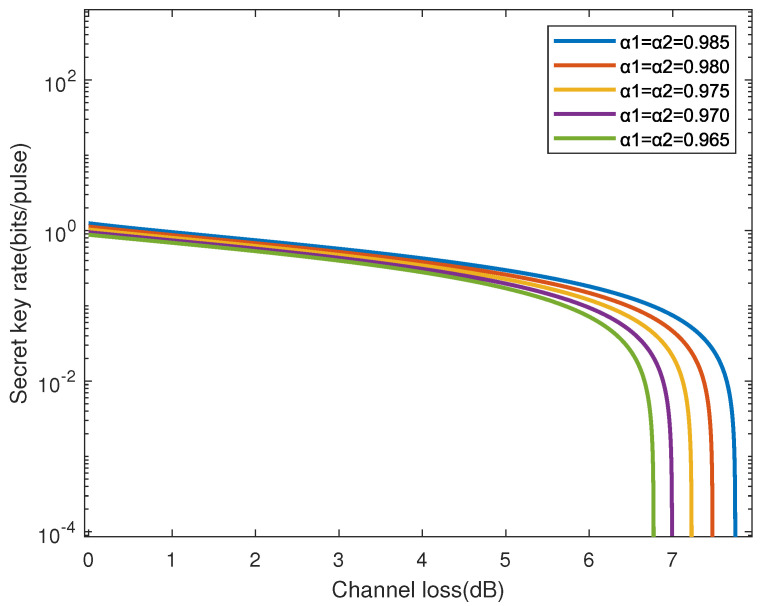
Secret key rates for different Bogoliubov coefficients $\alpha_{I}$, $\alpha_{II}$ as a function of channel losses are shown. From top to bottom (right to left) are 0.985, 0.980, 0.975, 0.970, and 0.965, respectively. The other parameters: $V_{0}$ = 1.1, W = 1, ϵ = 0, and β=0.98.

Figure 4 illustrates the key variation in quantum key distribution (QKD) under different Bogoliubov coefficients $\alpha_{I}$, $\alpha_{II}$, considering the effects of thermal states and acceleration. Here, the Bogoliubov coefficients are determined by the acceleration, the width of mode function *L*, and the frequency Ω. Normally, α=0.985 and $\beta =4.51\times 10^{-11}$ when the acceleration α=0.1, L=2, and Ω=5. The figure shows that the secret key rate decreases as the channel losses increase. This indicates that in practical applications, the performance of the QKD system is affected by environmental factors causing signal attenuation.

In the case of identical channel losses, the key rate decreases more rapidly with the increase in the Bogoliubov coefficients $\alpha_{I}$, $\alpha_{II}$. It is also observed that when the channel loss is 0dB, the secret key rate is close to $10^{0}$ bits/pulse for all curves. However, the secret key rate decreases rapidly as the channel losses increase. Particularly after the channel losses exceed 6dB, the curves become steeper, indicating that the security of the QKD system may be threatened under high-loss conditions.

**Figure 5:**
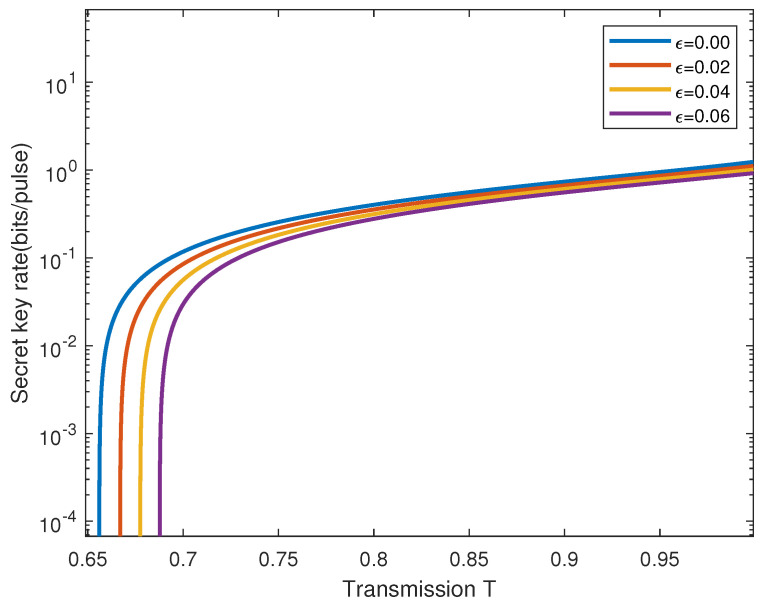
Secret key rate for different excess noise ϵ as a function of channel losses is shown. From top to bottom (right to left) are 0, 0.02, 0.04, 0.06, and 0.08, respectively. The negotiation efficiency is β=0.98.

Figure 5 illustrates the key variation in QKD under different levels of excess noise (ϵ), considering the effects of thermal states and gravity. The horizontal axis represents transmission T, while the vertical axis indicates the secret key rate per pulse (bits/pulse). The figure shows that the secret key rate per pulse gradually decreases as the T increases.

Specifically, when ϵ = 0, the blue curve represents the system without any additional noise. As ϵ increases to 0.02, 0.04, and 0.06, the system’s performance is defined by the red, yellow, and purple curves, respectively, showing the impact of different additional noise levels. It is observed that with increasing levels of excess noise, the system’s performance deteriorates more rapidly. This indicates that excess noise significantly impacts the QKD system, leading to a substantial decrease in the key rate.

## 5. Conclusions

This paper studied the performance of thermal-state continuous-variable quantum key distribution (CV-QKD) under the influence of gravitational effects. The study abandons the traditional use of pure Gaussian states, opting instead for Gaussian thermal states as the initial encryption resource. This choice is significant because thermal states more accurately represent real physical systems, which inherently contain a certain amount of noise. In the presence of gravitational effects, the security of the key rate is a critical issue. The paper calculates the covariance matrix of the resource state in the reference frame and analyzes the impact of relativistic acceleration on the performance of thermal-state quantum information protocols in realistic settings. Despite these potential challenges, the results of the paper indicate that the establishment of a secure key remains feasible even under the influence of gravitational effects. However, the paper acknowledges that addressing the issues of thermal-state CV-QKD under gravitational influence is a complex challenge. The interaction between thermal-state resources and gravity introduces a variety of factors that must be carefully considered. For example, the thermodynamic nature of the states introduces additional noise, which can be exacerbated by gravitational effects, potentially leading to a reduction in the key rate or increased susceptibility to eavesdropping.

Regarding the outlook, the paper anticipates that this area of research will be the subject of future studies, as it opens up new avenues for understanding the fundamental limits of quantum communication in the presence of gravitational phenomena. Researchers will need to develop new theoretical frameworks and experimental techniques to fully understand and mitigate the effects of gravity on CV-QKD. This may involve designing new protocols specifically tailored for operation in environments where gravitational effects are pronounced, such as near large masses or within satellite-based quantum communication networks. Ultimately, the knowledge gained from this research will demonstrate the potential of thermal-state CV-QKD protocols in satellite communications and may contribute to the development of more reliable and secure quantum communication systems under different gravitational conditions. For the experimental implementation, it shows great potential in terms of high anti-background noise capability, high key rate, and low power consumption by using heat sources instead of active modulation. Thermal-state CVQKD significantly reduced the system complexity and cost, and demonstrated strong robustness against background light noise. The experiments mostly use a 1550 nm wavelength laser, as it has the lowest loss in both the atmosphere and the optical fiber. And it can be achieved at room temperature. All in all, thermal-state CVQKD exhibits advantages in experimental implementation.

## Data Availability

Data can be obtained from the corresponding author.
